# Conversion of Unicompartmental Knee Arthroplasty to Total Knee Arthroplasty for Cement Allergy

**DOI:** 10.1016/j.artd.2024.101496

**Published:** 2024-10-11

**Authors:** Alexander Edelstein, Andrew Lachance, Coleman Cush, Jeffrey Lutton

**Affiliations:** a Department of Orthopaedic Surgery, Guthrie Robert Packer Hospital, Sayre, PA, USA; b Lewis Katz School of Medicine, Temple University, St. Luke’s University Health Network, Bethlehem, PA, USA

**Keywords:** Revision TKA, Cement Allergy

## Abstract

Despite the success of unicompartmental knee arthroplasty (UKA) and total knee arthroplasty (TKA), complications leading to loosening and eventual failure can arise. While infection, instability, and aseptic loosening are some of most common causes of UKA and TKA failure, one of the less common reasons is delayed hypersensitivity reactions. While most allergic reactions are hypersensitivity reactions to metal, hypersensitivity reactions to other materials used in the procedure, such as polymethylmethacrylate bone cement, have begun to gain more attention in recent years. In this case report, we explore the unique instance of a patient who required a revision of a cemented UKA to TKA due to severe pain likely caused by a confirmed polymethylmethacrylate allergy.

## Introduction

Despite the success of unicompartmental knee arthroplasty (UKA) and total knee arthroplasty (TKA), complications leading to loosening and eventual failure can arise. While infection, instability, and aseptic loosening are some of the most common causes of UKA and TKA failure [1], one of the less common reasons is delayed hypersensitivity reactions [2]. A delayed, or type IV, hypersensitivity reaction is a T-cell mediated reaction causing an inflammatory response [3]. In one study, delayed hypersensitivity reactions were the cause for 9.8% of revisions in patients with sensitivity to an implant component [4]. The most common culprit of delayed hypersensitivity reactions causing revision TKA surgery is metal particles from the articular surface of the femoral component [5,6]. Delayed hypersensitivity reactions to implants result in the occurrence of aggressive synovitis and severe pain, often accompanied by eczema [2,[4], [5], [6]].

While most allergic reactions are hypersensitivity reactions to metal [6], hypersensitivity reactions to other materials used in the procedure, such as polymethylmethacrylate (PMMA) bone cement, have begun to gain more attention in recent years [2,[5], [6], [7]]. Within bone cement, PMMA comes as a powder and acts as the polymer, benzoyl peroxide as the initiator, and a radio-opacifier. The liquid component contains methylmethacrylate as the monomer, as well as an accelerator (N, N-dimethyl para-toluidine) and a stabilizer (hydroquinone) [8]. PMMA, a key component of orthopaedic bone cement, has long been employed in joint arthroplasty procedures for its bonding properties and stabilizing prosthetic components [8]. Bone cement works as a “grout-like” material, in which its properties are not necessarily adhesive; rather, it acts as a space filler between the bone/implant interface, which in turn holds the implant against the bone [8]. Evidence suggests that delayed hypersensitivity reactions to PMMA may occur, which may lead to complications requiring a revision procedure. Histopathologic findings of periprosthetic tissues in these cases may demonstrate multinucleated giant cells with lymphoplasmacytic infiltration around the cement particles [7].

In this case report, we explore the unique instance of a patient who required a revision of a cemented UKA to TKA due to severe pain likely caused by a confirmed PMMA allergy.

## Case history

**Figure 1:**
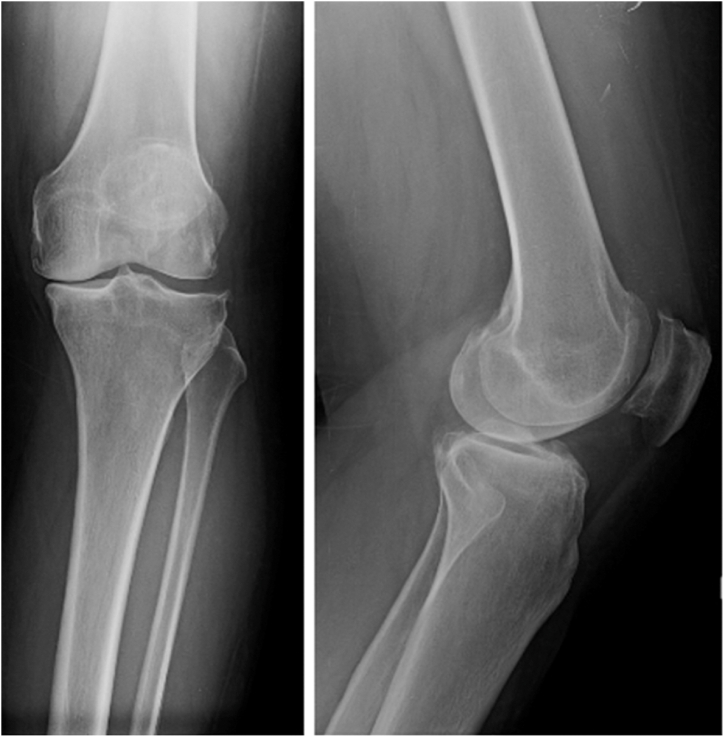
Anterior-posterior and lateral X-rays of the left knee prior to initial UKA.

**Figure 2:**
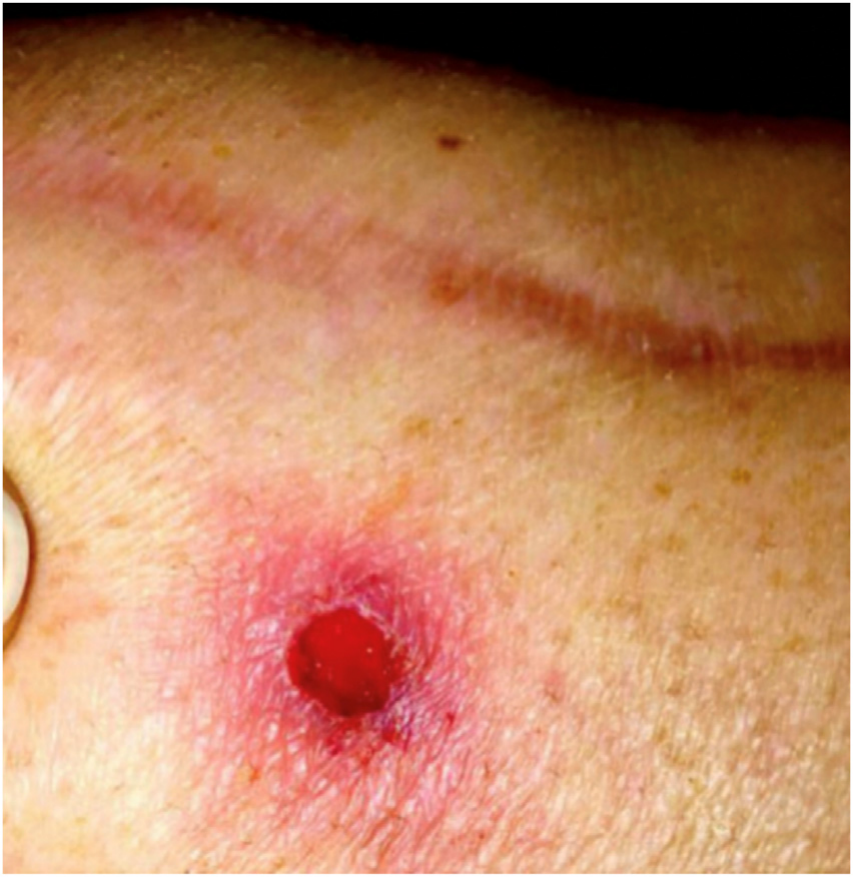
Demonstrates a small wound over the medial joint line, medial to the surgical incision.

**Figure 3:**
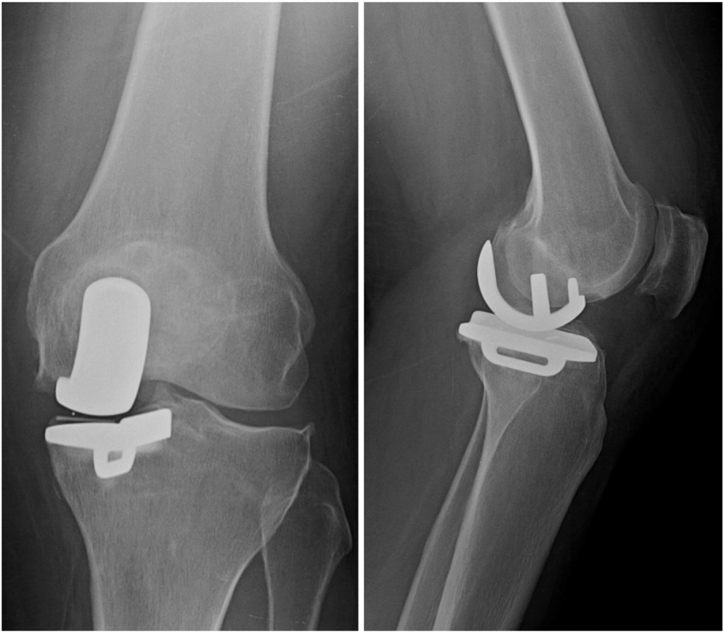
Anterior-posterior and lateral X-rays of the left knee at her 3-month postoperative appointment.

The patient was a 46-year-old female with a history of depression and fibromyalgia, which were controlled with selective serotonin reuptake inhibitors and endometriosis. She had a body mass index of 29.05 kg/m^2^, no prior allergy history, no history of tobacco use, and no history of narcotic use. The patient initially presented 6 years prior to the index procedure, complaining of pain over the medial aspect of her left knee. She stated that the pain was worse with walking and activity. She did not have a limp. She went to physical therapy, which worked for 3 years but then provided no relief. She received multiple corticosteroid and Synvisc (Sanofi Genzyme, Cambridge, MA) injections, both of which initially worked but then began to fail after 2 years. Throughout her course of conservative management, she did not use any opiates. She then underwent a left knee arthroscopy with partial medial meniscectomy of the body and the posterior horn for meniscal tears. She was also noted to have Kellgren and Lawrence grade 3-4 osteoarthritis in the medial compartment. She then underwent a left medial unicompartmental knee arthroplasty at the author’s institution for isolated medial compartment osteoarthritis using the Zimmer Biomet Oxford Partial Knee System **(**Zimmer Biomet, Warsaw, IN) (Fig. 1.) Postoperatively, she noted persistent pain of at least 4/10 at all times. In addition, at her 3-month postoperative visit, it was noted that she had developed a small punctate wound anterior to her medial joint space (Fig. 2.) An initial periprosthetic joint infection (PJI) workup was performed, with no elevation in her inflammatory markers. Her white blood cell count was 7.0 × 10^9^/L [3.6 × 10^9^/L-11 × 10^9^/L], erythrocyte sedimentation rate (ESR) was 16 mm/hr [0 mm/hr-20 mm/hr], and her C-reactive protein (CRP) was 0.9 mg/L [<1.00 mg/L]. There were no lucencies noted on her X-ray (Fig. 3). She noted that 1 month ago, she was found to have an infected tooth and was started on Clindamycin PO 150 mg twice per day. At this point, it was decided to aspirate her knee. Her cultures were negative, and the nucleated cell count of the aspirate was 998 cells/μL, with a synovial fluid appearance that was thin and bloody and not purulent. She had no fevers, chills, or other systemic signs of symptoms of infection. It is important to note that this aspiration was performed several weeks after she had started her PO antibiotics, which could have altered the results of the cell count and culture, though given her constellation of symptoms, it was not believed that she had a PJI. The antibiotics were continued for her dental infection, and she was told to continue them until her wound healed. This wound was treated by the wound care clinic and gradually resolved over time. The wound was treated with multiple debridements, Silvercel, and a dry sterile dressing. The wound took approximately 3 weeks to heal, and antibiotics were stopped after it had healed. At her 4-month follow-up, she noted that she had persistent “shooting pain” in her left knee, which she described as pain over the medial aspect of the knee with activity. Her knee range of motion was 0-120, and she had a normal gait. Physical exam demonstrated diffuse medial joint line pain. At this point she was using a fentanyl patch 75 mcg changed every 3 days for persistent pain. She was still taking her baseline medication for her fibromyalgia, and she stated that this pain felt different than her fibromyalgia pain.

At her 1-year postoperative appointment, she continued to complain of persistent “shooting pain” in the left knee. No additional wounds had developed at or around the surgical site, and the prior wounds had healed without complications. On exam, she was found to have persistent diffuse medial joint line tenderness similar to her 4-month follow-up appointment. She was recommended to restart physical therapy, which did not help. Three months later, she again presented with the same symptoms. The patient then had a metal allergy panel performed because she did not appear to have a PJI or instability and a reason for her pain could not be discerned. The allergy testing was performed by a dermatologist using a metal allergy patch, which was positive only for methyl methacrylate with severe reactivity. She was negative for nickel, cobalt, aluminum, chromium, iron, titanium, vanadium, and zirconium. She was then lost to follow-up for 2 years.

**Figure 4:**
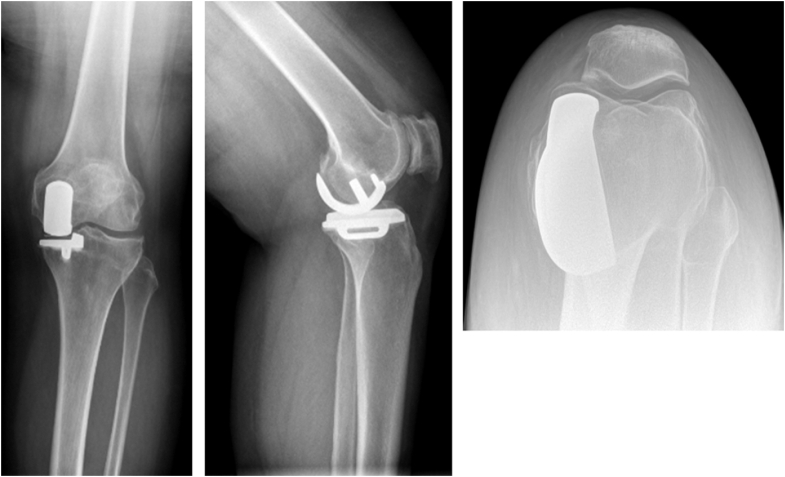
Anterior-posterior, lateral, and sunrise X-rays of the left UKA prior to revision.

She returned to clinic 3 years after her initial surgery with gradually worsening pain in her left knee. A repeat PJI workup was performed, with her white blood cell count, ESR, and CRP all within normal limits. There were no lucencies noted on her X-ray (Fig. 4.) Her only positive finding throughout this workup was her positive patch allergy test to methyl methacrylate, so she was scheduled for a UKA to TKA revision using press-fit components.

**Figure 5:**
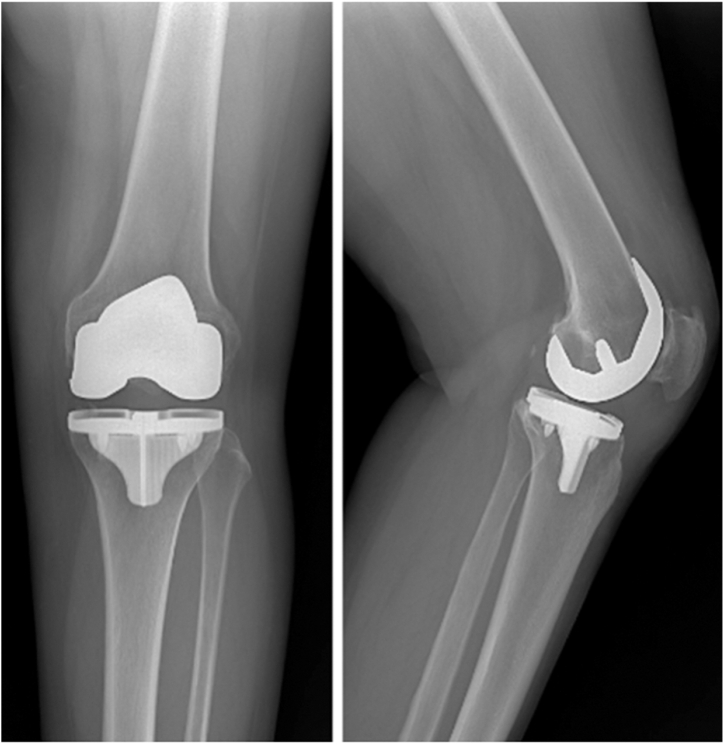
Anterior-posterior and lateral X-rays of the left TKA revision 6 weeks postoperatively.

The patient underwent robotic-assisted UKA to TKA conversion approximately 44 months after her index procedure, using cementless press-fit components (Stryker, Mako, Kalamazoo, MI) (Fig. 5.) The robot was used in this case for multiple reasons: easy to plan the procedure with a preoperative CT, can make accurate bony cuts to minimize bone loss, and surgeon preference. For this case, the same incision as her UKA was used and extended proximally and distally. The implants were removed using osteotomes to break up the implant-cement interface. The majority of the cement was removed during the bony cuts, and the rest of the cement was removed using curettes and osteotomes. There were only a few very small bony defects that were adequately bone grafted. The bone was tested intraoperatively to make sure that press-fit implants would be appropriate, as that is the preference of the surgeon. A press-fit knee with a size 13 cruciate-retaining polyethylene component was implanted. The patient tolerated the procedure well with no acute complications. An intraoperative tissue culture was sent, demonstrating chronic inflammation with no evidence of infection.

Postoperatively, she was unable to attend physical therapy for 2 months due to personal reasons that she did not explain and felt her knee was stiff and sore. At 2-months postoperative, her range of motion (ROM) was 5-80. Her surgical incision had healed well, and she did not develop any additional wounds. She was brought to the operating room for a manipulation under anesthesia, during which her range of motion was improved from -2-90 degrees to -2-130 degrees using slow gentle pressure. She was set up for physical therapy to start soon after.

**Figure 6:**
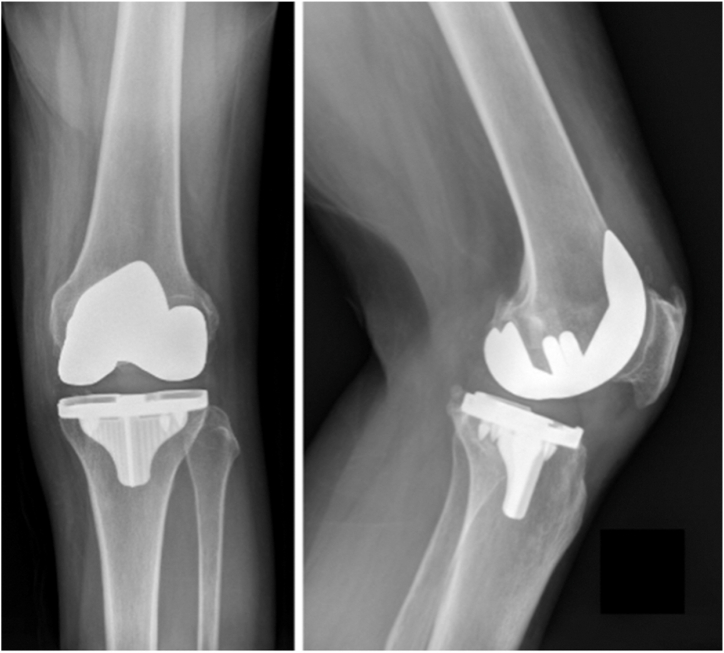
Anterior-posterior and lateral X-rays of the left TKA revision 18 months postoperatively.

By 4 weeks postmanipulation, she had decreased pain compared to preoperative, and by 3 months postmanipulation, her ROM was 5-110 with almost complete resolution of her pain. At 18 months postrevision, her ROM was unchanged, and the implant was stable on X-ray (Fig. 6.)

## Discussion

The problem of a painful knee after UKA or TKA can be a complex one. We report the first case of PMMA hypersensitivity in a UKA, successfully managed with press-fit robotic-assisted TKA. While there are no series discussing revision of a cemented UKA, there are series describing revisions of cemented TKAs to cementless TKAs due to an allergy. There are several different allergens that may cause a patient to require a revision total knee arthroplasty. A case series by Pahlavan et al [9] describes a series of 7 patients with 8 painful TKAs that required revision due to various allergies. Of the 8 knees requiring revision, all 8 had complaints of pain, 5 had complaints of chronic effusions, 4 had complaints of arthrofibrosis, and 3 had complaints of instability. Infectious workups were all negative except in one patient with an elevated CRP. Allergens present included cobalt, nickel, benzoyl peroxide, bone cement monomer, and bone cement powder. All revisions were done with custom cementless implants. Pain, ROM, and patient-related outcome scores were significantly improved in all patients after revision. PMMA is not the only component of bone cement that can cause a delayed hypersensitivity reaction. There are several case reports/series of patients with confirmed hypersensitivity to benzoyl peroxide [[10], [11], [12], [13]]. In these reports, the patients all experienced similar symptoms, most commonly significant pain, which resolved after cementless revision. There is also one case report of a patient who developed a pseudotumor secondary to methyl methacrylate sensitivity [14]. The patients in these case reports and the above series followed a similar pattern to the patient in this case report, with a presenting symptom of pain with a negative infectious workup and subsequent significant improvement in pain and ROM after revision with cementless components.

Existing literature highlights the multifaceted nature of delayed hypersensitivity reactions in orthopaedic procedures, involving not only PMMA but also metal components. A recent study by Whiteside et al [5] reported successful outcomes in patients revised with custom porous-coated ceramic femoral components. These 5 patients had a mix of sensitivities to nickel (Ni), molybdenum (Mo), and PMMA. All 5 patients had significantly increased Knee Society scores at their 3-year and most recent postoperative visits compared to their preoperative visits. This case series did also evaluate 7 patients with either Ni or Mo allergies who underwent a revision with a cemented ceramic-coated femoral implant and similarly found significantly increased Knee Society scores at their 3-year and most recent postoperative visits compared to their preoperative visits. There were also 4 patients who had a mix of various metal allergies and were revised at an outside institution with a cobalt-chrome femoral implant, and those patients had no significant change in Knee Society scores at their 3-year and most recent postoperative visits compared to their preoperative visits.

## Summary

It is important to consider the extensive list of differentials when evaluating patients with a painful TKA. While the most common modes of TKA failure are infection, instability, and aseptic loosening, there are many other potential less common etiologies. These can include, but are not limited to arthrofibrosis, central sensitization, component malposition, hypersensitivity reaction, patellar maltracking, scar neuromas, tendinitis and bursitis of the pes anserinus and biceps femoris tendons, complex regional pain syndrome, and peroneal nerve irritation [15,16]. All evaluations should start with a physical exam and X-ray, followed by bloodwork including an ESR, CRP, and complete blood count with differential. If these lab values are elevated, aspiration of the joint is the next step to rule out infection, looking at nucleated cell count, gram stain, and culture. Alpha-defensin can also be a useful tool in diagnosing PJI [17]. A bone scan may also be helpful in identifying aseptic loosening not seen on X-ray [18]. CT scan may be useful in the setting of a painful TKA to evaluate for areas of osteolysis and fracture and can be used to determine if there is an element of component malposition [19]. While a delayed hypersensitivity reaction is one of the less common reasons for TKA failure, it should be considered, and comprehensive allergy testing should be part of the workup once other more common causes of pain are ruled out. We demonstrate a successful conversion from a cemented UKA to a well-balanced robotic-assisted, press-fit TKA.

## Conflicts of interest

The authors declare there are no conflicts of interest.

For full disclosure statements refer to https://doi.org/10.1016/j.artd.2024.101496.

## Informed patient consent

The author(s) confirm that written informed consent has been obtained from the involved patient(s) or if appropriate from the parent, guardian, power of attorney of the involved patient(s); and, they have given approval for this information to be published in this case report (series).

## CRediT authorship contribution statement

**Alexander Edelstein:** Writing – review & editing, Writing – original draft, Conceptualization. **Andrew Lachance:** Writing – review & editing, Writing – original draft. **Coleman Cush:** Writing – review & editing, Writing – original draft. **Jeffrey Lutton:** Supervision.

## Appendix A Supplementary data

Conflict of Interest Statement for Cush

Conflict of Interest Statement for Edelstein

Conflict of Interest Statement for Lachance

Conflict of Interest Statement for Lutton
